# Ultrasound-Induced New Cellular Mechanism Involved in Drug Resistance

**DOI:** 10.1371/journal.pone.0048291

**Published:** 2012-12-19

**Authors:** Mariame A. Hassan, Yukihiro Furusawa, Masami Minemura, Natalya Rapoport, Toshiro Sugiyama, Takashi Kondo

**Affiliations:** 1 Department of Radiological Sciences, Graduate School of Medicine and Pharmaceutical Sciences, University of Toyama, Toyama, Japan; 2 Department of Pharmaceutics and Industrial Pharmacy, Faculty of Pharmacy, Cairo University, Cairo, Egypt; 3 Department of Gastroenterology and Hematology, Graduate School of Medicine and Pharmaceutical Sciences, University of Toyama, Toyama, Japan; 4 Department of Bioengineering, University of Utah, Salt Lake City, Utah, United States of America; University of Colorado, United States of America

## Abstract

The acoustic effects in a biological milieu offer several scenarios for the reversal of multidrug resistance. In this study, we have observed higher sensitivity of doxorubicin-resistant uterine sarcoma MES-SA/DX5 cells to ultrasound exposure compared to its parent counterpart MES-SA cells; however, the results showed that the acoustic irradiation was genotoxic and could promote neotic division in exposed cells that was more pronounced in the resistant variant. The neotic progeny, imaged microscopically 24 hr post sonication, could contribute in modulating the final cell survival when an apoptotic dose of doxorubicin was combined with ultrasound applied either simultaneously or sequentially in dual-treatment protocols. Depending on the time and order of application of ultrasound and doxorubicin in combination treatments, there was either desensitization of the parent cells or sensitization of the resistant cells to doxorubicin action.

## Introduction

Multidrug resistance (MDR) is a unique inherent or induced system for protection by which cancer cells can experience reduced cytotoxicity in response to a wide range of chemotherapeutics. The non-specificity of this system, that is acquiring cross resistance to various unrelated drugs, undermines the outcomes of chemotherapy [1]. MDR comprises different mechanisms, the most common of which involves the reduction of intracellular drug accumulation. This occurs through the expression of membrane proteins that can extrude the internalized drug molecules before they can even reach the cytoplasm [2]. These proteins are energy-powered transporters belonging to the adenosine triphosphate (ATP)-binding cassette (ABC) superfamily proteins. Mammalian P-glycoprotein (P-gp) was the first identified member of this family and is present at varying levels in every human tissue [3]. As mentioned earlier, the ABC transporters pump a broad spectrum of substrates which hardly share a common structural lead or action. However, the knowledge of membrane composition and how it impacts the internalization of exogenous molecules into cells suggests that pump substrates might share a degree of lipophilicity as a common trait. In fact, all P-gp substrates are lipophilic compounds which can readily cross the cell membrane in absence of the efflux proteins. Based on this, it might be also expected that P-gp-expressing cells might have different membrane properties to provide an optimal phase balance for their efficient functioning [4].

If the intracellular concentration of P-gp substrates is a result of the equilibrium attained between drug uptake by passive diffusion and drug efflux by these multi-drug transporters, then, modulating one of these two factors can result in increasing the intracellular accumulation of these compounds. Not only is this step indispensable in overcoming resistance attributable to P-gp expression, but also indispensable in tumor cells possessing other resistance pathways (e.g. drug inactivation). In general, increasing the intracellular concentration of drugs serves in surpassing the threshold of cells to reverse their toxicity efficiently.

The basic strategies in Pgp-mediated MDR reversal sought the inactivation of the efflux proteins, either directly through the use of inhibitors [5] or indirectly through ATP depletion or membrane fluidization [6], [7]. Projecting the knowledge of Ultrasound (US) interactions with biological systems on MDR reversal, we can predict numerous scenarios of sensitization. The most prominent effect of US is its ability to (transiently) permealize cell membranes to P-gp substrates through sonoporation [8]. Also, US-induced hyperthermia, due to the partial absorption of acoustic energy, can increase the accumulation of drugs probably due to (transient) membrane fluidization that might affect the functioning of the efflux pumps [9], [10]. Acoustic effects are not limited to cellular membranes; however, they extend to intracellular targets including mitochondria, endoplasmic reticulum and the nuclear territory. The impact of US hits on intracellular targets manifests as increased intracellular oxidative stress, induction of apoptosis [11], [12], [13], alteration in gene expression levels, and DNA damage [14], [15], [16]. Although these manifestations correlated with increased cell killing in many studies, reflecting the potential of US as an adjuvant tool in cancer eradication and further supporting the rationale of employing US in MDR reversal, there were occasions in which the enhancement of cell killing was not satisfactory, especially for solid tumor-derived (adherent) cancer cell lines [17], [18]. The decade-old studies on the use of US in MDR reversal showed in some cases higher sensitivity of drug-resistant cells to US exposure [8], [19], [20]. This interesting and important, and yet unexplained, finding never correlated to the number of studies on this approach nor to the body of knowledge accumulated over these years on the underlying mechanisms. We have also noticed that the relatively successful trials were reported on a limited number of cancer cell lines (e.g. human hepatocarcinoma and ovarian carcinoma). The somewhat stymied progression in this issue implies that the final outcome has not been always encouraging or plausible.

In an effort to define the impact of US in MDR, we planned this study to evaluate the differential sensitivities of drug-sensitive uterine sarcoma cell line (MES-SA) and its doxorubicin (Dox)-resistant variant (MES-SA/DX5 cells) as a new model cell line to US exposure from different analytical perspectives. Despite the observed higher sensitivity of resistant cells to US exposure being consistent with previous studies, our results came to unveil new hazardous consequences of the application of genotoxic low-intensity pulsed ultrasound in cancer therapy. We also extended the scope of the work to investigate the implication of our findings when US is combined with Dox under various combination protocols. This study introduces a novel effect of genotoxic US and highlights the significance of temporal settings in combination treatments. The presented results necessitate exploring new roads in the future to understand the interaction between US and biological systems, especially when it approaches a genetic level, in support with the statement of Yu et al. that “ultrasound was a “two-edged sword” [19].

## Materials and Methods

### Cell line and cell culture

MES-SA human uterine sarcoma cells [21] and its multidrug resistant phenotype (MES-SA/DX5) [22] were obtained from American Type Culture Collection (ATCC; Manassas, VA). The resistance of MES-SA/DX5 cells is attributed to the expression of P-gp [23]. Cells were grown in McCoy's 5A medium (Gibco BRL, Grand Island, NY) supplemented with 10% fetal bovine serum and 1% antibiotic mixture. For splitting, cells were detached from the culture dishes using a non-enzymatic cell dissociation solution (Sigma-Aldrich). MES-SA/DX5 was treated with 500 nM of Dox for 1 hr once every 8 passages to maintain their Dox resistance [24]. Also, the positive controls from each experiment were compared to monitor the resistance level and exclude outlier sets. For experiments, cells were seeded in 3.5-cm culture dishes (Corning Inc., Corning, NY, code # 430165) at a density of 1×10^6^ cells 24 hr before experiments unless otherwise mentioned. Before treatment, the samples media were replaced with warm fresh air-saturated media up to 2 ml final volume/dish.

### Acoustic setup and sonication

A 1 MHz medical acoustic generator (Sonicmaster ES-2, OG Giken Co., Ltd., Okayama, Japan) was used in this study [25], [26]. The detailed properties of the setup are given in Table 1. For sonication, the transducer was clamped with its surface facing upward and coupled with culture dishes placed above it with partially degassed water. This arrangement results in standing waves, and thus the real acoustic pressure should be expected to be higher than estimated in Table 1. Sonication was carried out at intensities of 0.2, 0.3, 0.4 and 0.5 W/cm^2^ at 10% duty cycle (DC) and lasted for 60 s at room temperature for each sample. The range of the intensities chosen presented regions below and above the cavitational threshold of this setup which was shown to occur at 0.3 W/cm^2^ [27]. Exposed cells were allowed to incubate for further 24 hr unless otherwise mentioned. For analyses, cells were collected quantitatively, washed twice and finally suspended in cold phosphate buffered saline (PBS). Samples were kept on ice during handling to minimize progression of biochemical processes.

**Table 1:** Properties of the acoustic unit.

Transducer	planar - 50 mm in diameter				
**Central frequency (MHz)**	1.0				
**Device - indicated intensity (W/cm^2^)** *	0.1	0.2	0.3	0.4	0.5
**I_SATA_ at 10% DC (W/cm^2^)** †	0.048	0.072§	0.081§	0.092§	0.105§
**Output pressure (MPa)** ‡	0.061	0.105	0.132	0.144	0.146
**Operation modes**	Continuous wave mode – Pulsed wave mode				
**Duty Cycle (DC, %)**	10, 20, 30, 50 & 100				
**Pulse Repetition Frequency (PRF, Hz)**	100				

* The intensity ranges from 0.1–2 W/cm^2^ at an increment of 0.1 W/cm^2^.

† The output intensity measured by an ultrasound power meter (UPM-DT-10E, Ohmic Instrument Co., Easton, MD) from the transducer surface and divided by the effective surface area of the transducer. Attenuation due to the culture dish was practically negligible (<10%).

‡ The output pressure calculated from the equation *I = P^2^/ρc*, where (*I*) is the intensity [W/m^2^], (*P*) is the acoustic pressure, (*ρ*) is the density of the medium and (*c*) is the speed of sound in the medium.

§ The intensity used in sonication experiments.

### Determination of cell viability

Equal aliquots of cell suspensions were inoculated into 96-well plate containing 100 µl of fresh pre-warmed medium/well. 10 µl of the tetrazolium salt WST-8 (Cell Counting Kit-8 (CCK-8); Dojindo Molecular Technologies, Inc., Rockville, MA) were added to each well and incubated for 4 hr after which the color density of the soluble formazan dye product was measured at 450 nm. Each assay was performed in duplicates and McCoy's 5A medium was used as a blank control. Untreated controls were taken as 100% viability.

### Cell counting assay

The number of total cells in each sample was determined using a cell coulter counter (Coulter® Z1, Coulter Electronics Ltd., England) [28]. Equal aliquots of cell suspensions were mixed with Isoton II diluent in Z1 cups according to the manufacturer's recommendations and then placed on the measuring platform for counting. Cells equal to or larger than the aperture opening were counted automatically. Cell counts were calculated based on control that was taken as 100% cells.

### Flow cytometric analysis

For the detection of phosphatidylserine (PhS) externalization as a marker of early apoptosis, and membrane integrity as an end point for cell death, portions of the cell suspensions were incubated with FITC-labeled Annexin V and propidium iodide (PI) from Apoptosis Detection kit (Immunotech, Marseille, France), respectively, according to the manufacturer's instructions. Cells were then injected into a flow cytometer (Epics XL, Beckman Coulter, Miami, FL) and the percentage of cells with single or double staining was obtained from counting 10,000 events per sample.

### Assessment of H2AX phosphorylation

Cells were sonicated at different intensities and then incubated for 15 min before they were collected quantitatively and fixed with 70% cold methanol overnight. Cells were permealized for 30 min at room temperature with 0.05% Tween/PBS containing 2% bovine serum albumin to block non-specific binding. Cells were then reacted sequentially with the primary monoclonal antibody (mAb) anti-phospho-H2AX S139 (γH2AX) (Upstate Biotechnology, Lake Placid, NY) and the secondary antibody Alexa Fluor 488 anti-mouse F (ab′) IgG (Cell Signaling Technology, Beverly, MA) for at least 1 hr for each antibody before they were analyzed flow cytometrically [26].

### Cell cycle analysis

Quantitatively harvested control and treated cells were fixed with 70% pre-chilled ethanol for at least 2 hr. Then they were re-suspended in PBS containing RNAase (Nacalai Tesque, Kyoto, Japan) at a final concentration of 0.25 mg/ml/1×10^6^ cells for 30 min at room temperature. Cell pellets were finally suspended in 50 µg/ml PI/PBS staining solution and incubated in dark at 4°C for 20 min before flow cytometric analysis. Cell cycle analysis was performed 1 hr after sonication and 24 hr later.

### Microscopic observation

After 24 hr post sonication, cells nuclear content and plasma membranes were stained by Hoechst 33342 (Sigma-Aldrich) and Alexa flour-488 – conjugated wheat germ agglutinin (WGA; Invitrogen, CA), respectively, for subsequent microscopic observation. After thorough washing with cold PBS, the attached cells were simultaneously double stained with Hoechst 33342 and WGA at a final concentration of 2 µg/ml of 2% paraformaldehyde (PFA)/PBS each for 20 min. Cells were immediately examined under a fluorescence microscope (Nikon Eclipse TE300; Nikon, Tokyo, Japan).

### Ultrasound – Doxorubicin dual treatment protocols

Doxorubicin HCl (Dox), purchased from Sigma-Aldrich, was dissolved in PBS at a concentration of 100 µM. The stock solution was sterilized by filtration through a 0.22-µm filter and stored in aliquots at −20°C until use. In dual treatment protocols, Dox was added at a final concentration of 1 µM, the dose corresponding to the maximum apoptotic induction based on dose-dependent DNA fragmentation analysis (according to the method of Sellins and Cohen [18]). Two sets of protocols were carried out based on the time of application of each treatment; (a) **simultaneous-treatment protocols**, in which both Dox and US were applied simultaneously and DOX remained in the medium for 24 hr until measurements, (b) **sequential-treatment protocols**, in which either Dox or US was applied in the first day followed by the application of the other in the second day. Table 2 summarizes the experimental procedures in each set. For all the protocols listed, cells were seeded in 3.5-cm culture dishes (Corning Inc.) at a density of 1×10^6^ cells 24 hr before experiments. On the next day, media were replaced with warm fresh air-saturated media up to 2 ml final volume and handled according to Table 2. In cases where cells were to be incubated with Dox for a short period (60 min), only 1 ml of Dox-containing air-saturated medium was used for the pre-treatment followed by the addition of another 1 ml before acoustic exposure.

**Table 2:** A summary of the experimental procedures in dual-treatment protocols.

*(A) Simultaneous - Treatment Protocols*				
Sample	First Treatment	Incubation Period	Second Treatment	Incubation Period
**Control**	-	-	-	-
**US**	US	24 hr	-	-
**Dox**	Dox	24 hr	-	-
**US/Dox**	US	-	Dox	24 hr
**Dox/US**	Dox	-	US	24 hr
**Dox 60**	Dox	1 hr	US	24 hr

*(B) Sequential - Treatment Protocols*				
**Control**	-	-	-	-
**US 48**	US	24 hr	(q.w.)	24 hr
**Dox 48**	Dox	24 hr	(q.w.)	24 hr
**US - Dox**	US	24 hr	(q.w.)/Dox	24 hr
**Dox-US**	Dox	24 hr	(q.w.)/US	24 hr

**US**: Exposure of cells to 0.4 W/cm^2^ at DC 10% & PRF 100 Hz for 60 s.

**Dox**: Addition of Dox at 1 µM final concentration.

**q.w**.: quantitative wash without cell loss.

### Statistical analysis

Experiments were performed in independent triplicates and each data point is the average of two separately sonicated dishes. All results are displayed as mean ± standard error of the mean (SEM). Tests of significance were performed using one-way analysis of variance (ANOVA) followed by Tukey post-hoc test for multiple comparisons (α = 0.05) with p<0.05 considered to be statistically significant. Unpaired t-student test (two-tailed) was employed when two groups were to be compared with p<0.05 considered to be statistically significant. Pearson's correlation coefficient was used to test the correlation among data sets obtained. All tests were performed using GraphPad Prism 5 (GraphPad Software; San Diego, CA).

## Results

### Assessment of ultrasound-induced cell killing

**Figure 1:**
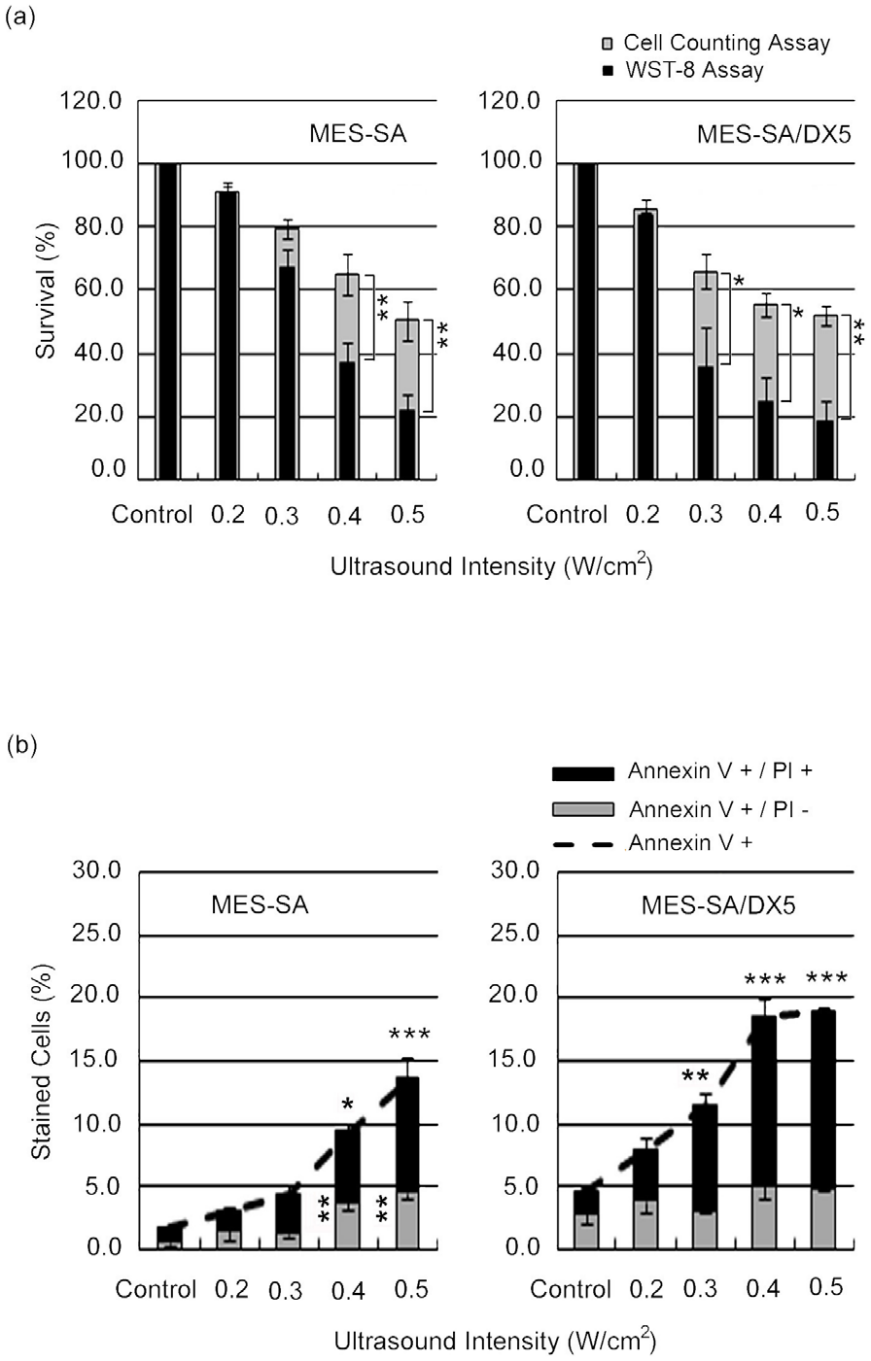
Ultrasound-induced cell killing in MES-SA and MES-SA/DX5 cells 24 hr post exposure to different acoustic intensities. (a) Cell viability assessed by WST-8 and cell counting assay. Asterisks (*) indicate the statistical significance of the difference between the absolute percentages obtained from WST-8 and cell counting assays at one intensity for one cell line. (b) Flow cytometric analyses for FITC-labelled Annexin V and PI staining. Vertically-aligned asterisks indicate the statistical significance of Annexin V (+)/PI (−) cells, whereas horizontally-aligned asterisks indicate the statistical significance of Annexin V (+)/PI (+) cells in comparison to control. Data points are presented as mean ± SEM.

As shown in Figure 1a, the exposure of MES-SA cells and its drug resistant variant to US at different intensities showed that the cell survival 24 hr post sonication measured by WST-8 assay and cell counting decreased in an intensity-dependent manner for both cell phenotypes. Table 3 shows that for both tests, MES-SA/DX5 cells yielded lower survival rates compared to MES-SA cells at almost each treatment condition, however, statistical significance was not attained except at 0.3 W/cm^2^, the intensity representing the cavitational threshold in this setup [27]. Despite the good correlation between the trends indicated by the WST-8 viability assay and cell counting results (Table 4), there were repeatable differences between the absolute values measured by the two techniques showing statistical significance at 0.4 W/cm^2^ for MES-SA cells and 0.3 W/cm^2^ for MES-SA/DX5 cells. These differences between WST-8 and cell counts results indicate the presence of cells that are equal to or larger than control cells which do not contribute to viability as measured by WST-8 assay. The lower threshold for this phenomenon in MDR cells together with the tendency of MDR cells to show lower values in both tests at 0.3 and 0.4 W/cm^2^, may suggest that a new US response takes place earlier in the MDR cell line.

**Table 3:** A summary of the differential responses of MES-SA and MES-SA/DX5 cells to US treatment.

		Treatment				
		Control	0.2 W/cm^2^	0.3 W/cm^2^	0.4 W/cm^2^	0.5 W/cm^2^
**WST-8 Assay (%)**	**MES-AS**	100.0±0.0	91.1±2.7	66.8±5.9*	37.1±6.6	22.2±5.2
	**MES-SA/DX5**	100.0±0.0	83.7±0.8	35.9±12.6	24.3±7.9	18.2±6.6
**Cell Counting (%)**	**MES-AS**	100.0±0.0	91.0±1.6	79.5±3.2*	65.1±6.6	50.3±6.3
	**MES-SA/DX5**	100.0±0.0	85.5±3.2	65.9±5.5	55.3±3.7	52.0±3.2
**Annexin V +/PI − cells (%)**	**MES-AS**	0.6±0.2	1.6±0.8	1.3±0.3	3.8±0.6	4.6±0.4
	**MES-SA/DX5**	2.9±0.9*	3.9±1.0	3.1±0.2*	5.1±0.9	4.9±0.3
**Annexin V +/PI + cells (%)**	**MES-AS**	1.1±0.10	1.5±0.26	3.2±0.60	5.6±0.76	9.0±1.77
	**MES-SA/DX5**	1.8±0.29	4.1±1.05*	8.2±1.11*	13.5±1.31*	14.0±0.40*

(*) denotes statistical significance between MES-SA and MES-SA/DX5 cells.

**Table 4:** The correlation coefficients (r^2^) between WST-8 assay and other tests employed to evaluate the extent of cell killing in uterine sarcoma cells.

	WST-8 Assay	
	MES-SA	MES-SA/DX5
**Flow cytometric analysis**		
**FITC-Annexin V+/PI−**	0.90	0.44
**FITC-Annexin V+/PI+**	0.94	0.9
**Cell Counting assay**	0.98	0.97
**Cell Cycle analysis (SubG1)**	0.77	0.88

The flow cytometric analysis indicated that the extent of membrane damage due to acoustic exposure, as perceived from the percentage of cells internalizing PI, was also significantly higher in MES-SA/DX5 cells (Figure 1b). Table 3 provides a summary of the responses of both phenotypes to different parameters of US under each analytical test. In what follows, we present most data for a power density of 0.4 W/cm^2^.

### Preferential exacerbation of resistant cells proliferation by ultrasound

**Figure 2:**
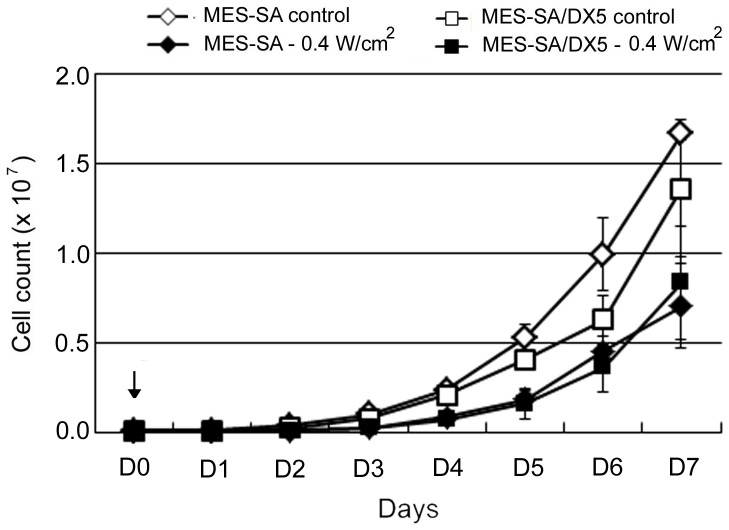
Proliferation of MES-SA and MES-SA/DX5 cells over 7 days (D1–D7) after sonication. Ultrasound was applied at an intensity of 0.4 W/cm^2^ on D0 (arrow). Cells were counted immediately after sonication and plated at a density of 1×10^5^ cells/dish. Data points are presented as mean ± SEM.

According to the cell provider's online information sheet, the doubling time of MES-SA/DX5 is longer than that of MES-SA cells indicating that MDR cells proliferate at slower rate compared to the parent cells [29] (Figure 2 – significance difference was observed at D2 and D3). Despite this, the assessment of cell counts over a 7-days period (D1–D7) showed that the there was no difference in the growth ratios (calculated as: cell count at D_n_/cell count at D_n-1_, where n is the day (D) number) between untreated cells for the two cell phenotypes under our culture conditions (r^2^ = 0.93). The average maximum growth ratio/24 hr for untreated MES-SA and MES-SA/DX5 cells was 2.7±0.2 and 2.8±0.3, respectively. Sonication at 0.4 W/cm^2^ caused insignificant immediate cell lysis (<10%) based on immediate cell counting after sonication (D0). Both sonicated cell phenotypes proliferated at a lower but similar rate than untreated cells consistent with the data of Kamaev and Rapoport [20] (Figure 2). This similarity between both cell phenotypes after sonication implies either that the parent cells were far more affected by US irradiation –which is in contrast to the viability results - or that the resistant cells have undergone accelerated increase in cell numbers. The inspection of the growth ratios revealed that only the sonicated parent cells fluctuated in proliferation with a maximum growth ratio of 3.7±0.5 observed on the fourth day following US exposure (D4), whereas the sonicated resistant cells displayed a steadier increase in growth ratio that peaked also on D4 at a growth ratio of 3.0±0.2. Moreover, when the growth ratio at Day 1 (D1) was calculated taking into consideration only the viable portion of seeded cells (based on WST-8 assay), the calculated growth ratio (0.52±0.1) was in the range of the observed value (0.6±0.2) for the parent cells, whereas the resistant cell phenotype showed a large difference between the calculated and observed values (0.27±0.1 vs 0.7±0.1, respectively) reflecting a dramatic enhancement in cellular proliferation even at D1.

### Nuclear budding in response to acoustic exposure

**Figure 3:**
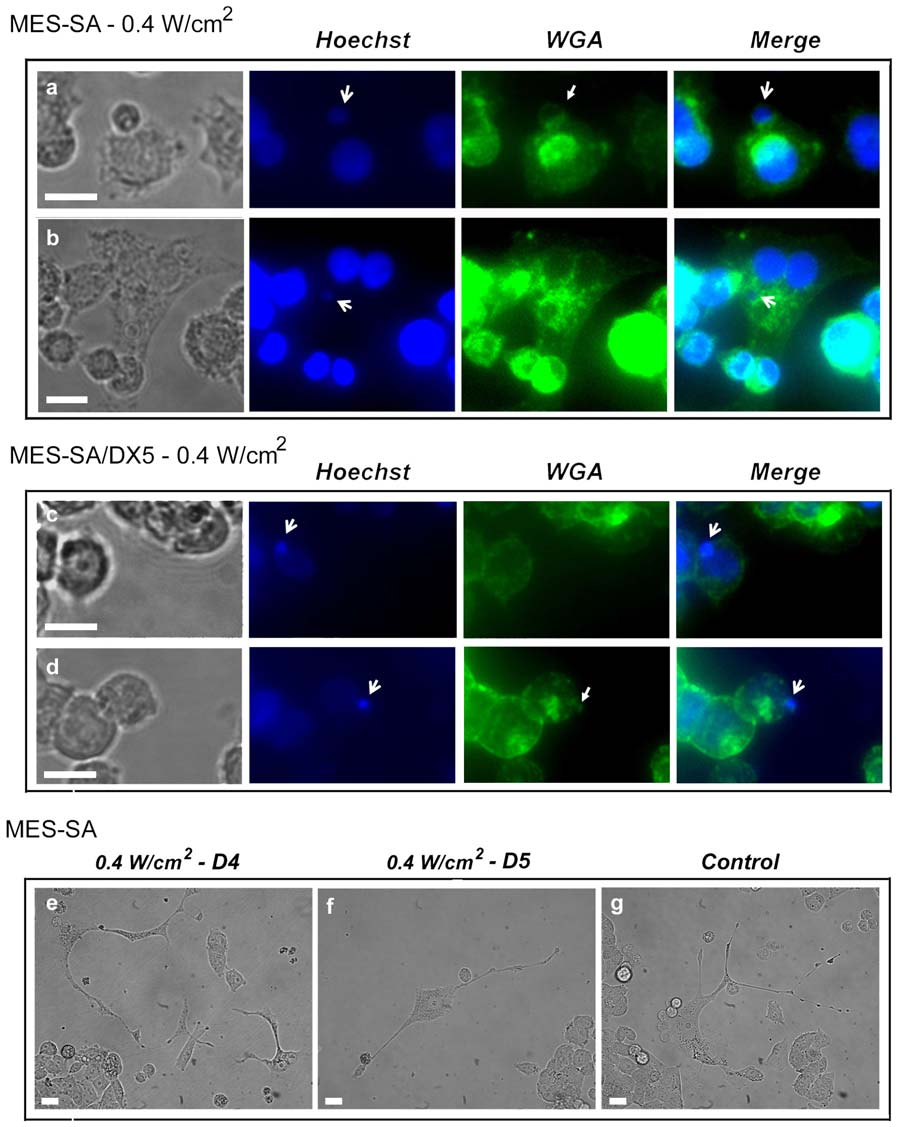
Fluorescent microscopy. Pictograms of MES-SA (a & b) and MES-SA/DX5 (c & d) cells 24 hr post sonication at 0.4 W/cm^2^. Cells were stained simultaneously with Hoechst 33342 and Alexa flour-488 – conjugated wheat germ agglutinin (WGA) in 2% paraformaldehyde (PFA)/PBS for 20 min followed by immediate observation. Cells show nuclear budding of genomic content (d) that is occasionally translocated through the cytoplasm (b) and emerges to form a small cell (Raju cell) (a). Two distinct forms of nuclear budding can be identified in treated MES-SA cells bearing similarity to neotic cytokinesis described by Rajaraman et al. [33], namely, (a) sequential cytokinesis type-2 perinuclear and (b) sequential cytokinesis type-1 pericellular, whereas the first form only was observed in treated MES-SA/DX5 cells. Open-head arrows indicate nuclear budding; closed-head arrows indicate the surrounding membranes of emerging cells. (e & f) Sonicated MES-SA cells showing multiple cytokinesis captured at the fourth (D4) and fifth (D5) days post exposure, respectively. (g) Control MES-SA cell with spontaneous cytokinesis. Cells (e–g) resemble in morphology neotic mother cells shown by Sundaram et al. [31]. Bars, 10 µm.

The microscopic examination of cells 24 hr after sonication at 0.4 W/m^2^ revealed the presence of different morphological features that can be approximately fitted to those reported recently in reference [30]. However, double staining of adherent cells with Hoechst 33342 and WGA showed the emergence of nuclear buds that were sometimes seen during translocation toward the cell membrane (Figure 3). Although the emergence of these buds occurred more frequently in sonicated MES-SA/DX5 cells, it was also observed in sonicated MES-SA cells, but at lesser frequency and in two distinct forms of emergence as shown in Figure 3 a & b. Some sonicated MES-SA cells captured four or five days post sonication showed morphological features similar to those reported by Sundaram et al. in a number of irradiated mouse- and human- derived cancers [31] (Figure 3 e & f). In some occasions, one or two similar cells were also observed in normal culture dishes indicating the spontaneity of the phenomenon (Figure 3 g).

### Induction of H2AX phosphorylation by ultrasound

**Figure 4:**
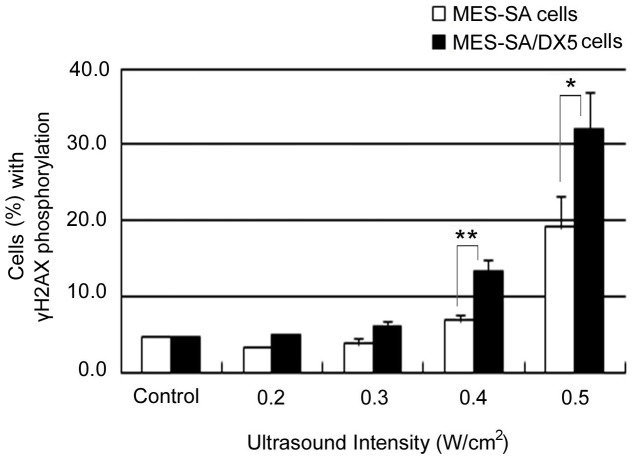
The extent of histone H2AX phosphorylation in MES-SA and MES-SA/DX5. Cells were fixed 15 min after exposure to ultrasound at different intensities. Cells were assayed flow cytometrically. Data points are presented as mean ± SEM. Asterisks (*) denote the statistical significance between MES-SA and MES-SA/DX5 at respective intensities.

The above findings suggest the reversion of cells, especially the MDR variant, to a mechanism of self-renewal which is very similar to neosis described by Sundaram et al., This mechanism works in the presence of a stimulus inducing genetic instability [31], therefore, we examined the occurrence of DNA damage by US through the detection of phosphorylated H2AX. The histone H2AX becomes phosphorylated on serine 139 to form the so-called gamma-H2AX (γH2AX) in response to induced DNA double strand breaks where the γH2AX foci act as platforms for spatiotemporal assembly of numerous proteins acting in response to DNA damage to form DNA checkpoints and facilitate DNA repair [32]. Here, we used the extent of H2AX phosphorylation as an indicator to DNA damage [26]. Measurements were performed fifteen minutes after US exposure at different intensities. The results in Figure 4 reveal an intensity-dependent increase in γH2AX which was significantly higher in MES-SA/DX5 cells confirming a higher degree of the ultrasound-induced DNA damage in resistant cells.

### Cell cycle distribution

**Figure 5:**
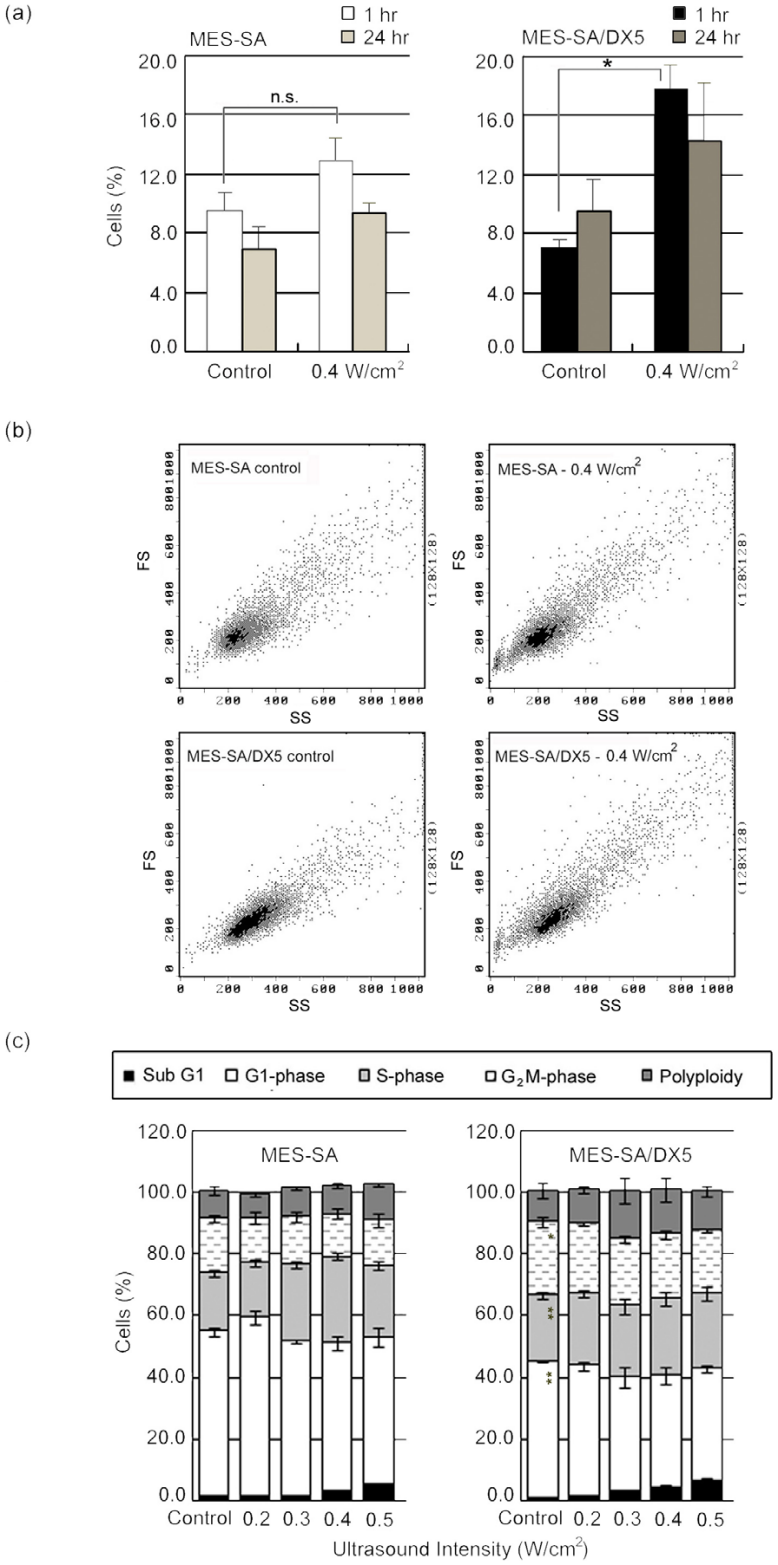
Cell cycle analysis of MES-SA and MES-SA/DX5 cells. (a) The fraction of polyploid cells detected 1 hr and 24 hr after sonication at 0.4 W/cm^2^. Data points are presented as mean ± SEM. Asterisks (*) denote statistical significance. (b) Flow cytometric dot plots of forward (y-axis; FS) versus side (x-axis; SS) scatter parameters showing the increase in cell size of MES-SA/DX5 cells 1 hr post sonication at 0.4 W/cm^2^. (c) Cell cycle analysis performed 24 hr post sonication at different intensities.

The cell cycle analysis revealed inherent differences between both phenotypes in fraction of cells in post-G1 phase which was 46.5±1.9% for MES-SA and 59.5±1.6% for MES-SA/DX5 cells. This difference reflects the genetic instability in DNA check points in MDR cells and further supports the susceptibility of MDR cells to abrupt neosis [33]. At one hour after US treatment, only the fraction of polyploid cells in resistant cells was significantly increased as compared to corresponding control. In accordance with the increase in polyploidy in MES-SA/DX5 cells, their scatter parameters indicated an increase in cell size (Figure 5 a & b).

At 24 hours post sonication, there were no significant changes in cell cycle phases suggesting a near recovery of acoustic effects [34], [35] (Figure 5c). However, the percentage of cells in the subG1 phase significantly increased with increasing acoustic intensity in both cell phenotypes, but never exceeded 7% at any condition. Therefore, it can be concluded that the immediate increase in H2AX phosphorylation could not have been a consequence of apoptotic DNA fragmentation (unlike that reported by Furusawa et al. 6 hr post sonication under similar conditions [36]), and that the decrease in cell survival after acoustic treatment is mediated by a mechanism other than apoptosis.

### The effect of ultrasound – doxorubicin dual treatments

**Figure 6:**
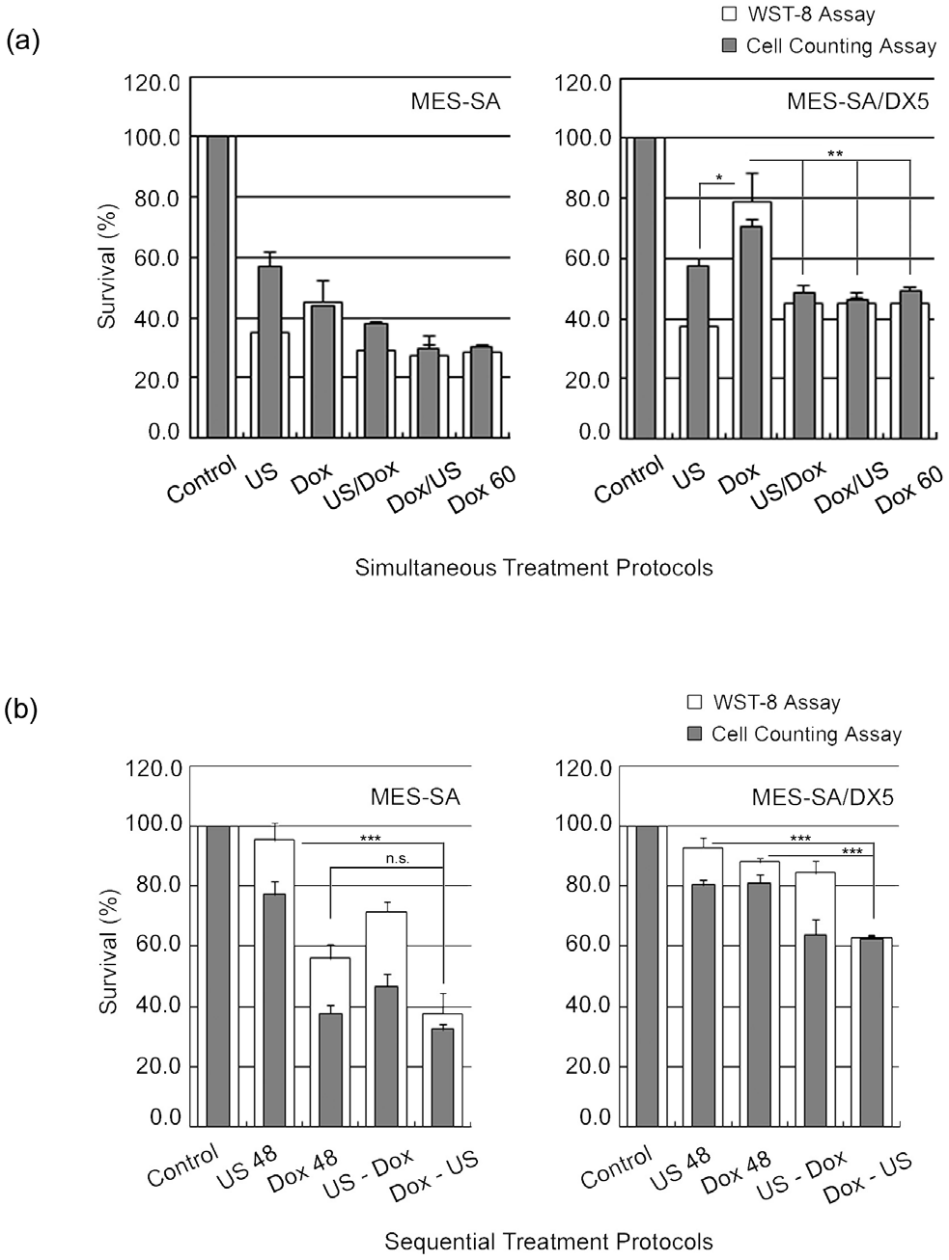
Dual treatment protocols. Cell survival following dual treatment protocols with doxorubicin (1 µM) and ultrasound (0.4 W/cm^2^). (a) Simultaneous treatment protocols. (b) Sequential treatment protocols. Data points are presented as mean ± SEM. Asterisks (*) denote the statistical significance of changes in cell survival following (Dox-US) protocol compared to (US 48) and (Dox 48) for each cell line. Following (US-Dox) treatment protocol, the cell survival of MES-SA cells decreased significantly compared to (US 48) but was insignificantly increased compared to (Dox 48). In MES-SA/DX5 cells, cell survival was unchanged. However, (US-Dox) protocol showed consistently large difference between the percentages obtained from WST-8 and cell counting assays indicating the presence of cells contributing to viability below the size threshold for detection by the cell counter.

- **Simultaneous-treatment protocols.** Figure 6 a shows that the cell survival (measured by WST-8) following different simultaneous combination protocols did not significantly change compared to that observed after US exposure alone for both cell phenotypes. However, if the data were compared to Dox treatment alone, only MES-SA/DX5 cells could exhibit a significant decrease in cell survival at all protocols. It is noteworthy that the difference between the absolute values obtained by the WST-8 and cell counting assay has diminished in all protocols except for (US/Dox) in MES-SA cells. The addition of Dox during (Dox/US) or immediately after (US/Dox) sonication did not alter the results of WST-8 assay. The acoustic intensity used here (0.4 W/cm^2^) is definitely above the cavitational threshold for this setup [25], [27], [36], and thus, the free radicals produced during sonication might have persisted indirectly in the medium to exert a delayed action rendering both conditions similar [11]. However, it is still reasonable to exclude a free-radical dependent cytotoxicity, being only likely with doses of Dox higher than that used in this study [25], [37], [38].
- **Sequential-treatment protocols.** A typical difference between physical and chemical treatments is demonstrated in Figure 6 b in which the acoustic effect was almost completely eliminated within 48 hr from exposure, whereas the effect of Dox persisted (in MES-SA cells only) despite washing out the drug. The accelerated recovery after US treatment observed here was likely due to the washing out of sonication medium [8]. In combination treatment, the (Dox-US) protocol led to decreased cell survival that was statistically significant in MES-SA/DX5, and abolished the difference between the absolute percentages obtained from the cell counting and WST-8 assays. This overlap of respective percentages from both tests under similar conditions may indicate that all counted cells contributed to measured viability. However, in other sequential treatment protocols, we observed “uncountable viability” where the survival fraction measured by WST-8 assay exceeded that measured by cell counter. This uncountable viability was accompanied by (i) a significant desensitization to Dox in MES-SA and a lack of effect in MES-SA/DX5 cells under (US-Dox) protocol, (ii) the observation of nuclear budding as early as 24 hr post sonication, and (iii) the evidence for US-induced stimulated growth in literature [8], [19], [39] as well as in our study. This “uncountable viability” may be caused by the presence of Raju cells that are beyond the detection threshold and, consequently, part of the counted non-viable large cells observed 24 after US exposure (Figure 1a & 6a) could represent neotic mother cells [40].

## Discussion

As shown by our data, the exposure of uterine sarcoma cell line to US resulted in an intensity-dependent growth inhibition in both the parent and Dox-resistant cell phenotypes. Generally, MES-SA/DX5 cells seemed to display more sensitivity to acoustic exposure in agreement with previous studies [8], [19], [20]. This selective sensitivity was more evident in the flow cytometric data of Annexin V (+)/PI (+) cells (Table 3), with a threshold for detecting significance at 0.4 W/cm^2^ for MES-SA cells and 0.3 W/cm^2^ for MES-SA/DX5 cells. On the other hand, the lack of appreciable percentages of DNA fragmentation (subG1 fractions and in DNA fragmentation assay according to the method of Sellins and Cohen [18] – data not shown) reflects that the straightforward interpretation of flow cytometric results for apoptosis is not fully appropriate in this case. The loss of membrane integrity - seen as PI uptake - can be autonomous at latter stages of cell death or induced by mechanical wounding of the membrane as that which may arise during US application [41], [42]. Cells expressing P-gp have been shown to possess higher membrane order for optimum functioning of the efflux pumps resulting in increased overall membrane rigidity [4], [43]. Also, the functioning of P-gp, as well as the sealing of the damaged parts of the membrane, requires the presence of ATP to provide energy. Considering these two features of MDR cells, namely, the higher membrane rigidity and the increased (basal) energy consumption, in the context of US-induced pores and the subsequent membrane sealing mechanisms [44], we may suggest that resistant cells may suffer from unfavorable conditions for membrane repair post acoustic exposure that manifests as increased uptake of normally impermeable dyes, such as Trypan blue [8], Sytox Green nucleic acid stain [20] and PI (Figure 1 b), or as a rapid drop in viability assayed by MTT assay [19] in comparison to parent cells. To further validate this conclusion, both cell lines were forced under similar pressure through a 30-gauge needle using 1-ml syringe [45]. MES-SA/DX5 cells showed more PI uptake after 1 hr incubation at 37°C, a time period that exceeds the required time for repairable damage to reseal [44]. Therefore, the more PI uptake observed in MES-SA/DX5 cells might have resulted from a failure in membrane repair and, as such, the membrane fragility against mechanical stressors is expected to undermine its protective role in absorbing the acoustic pressure allowing for easier transmission of the mechanical load through the cytoplasm, rendering the intracellular organelles, as well as the nucleus, accessible targets. This can further explain the higher extent of DNA damage found in the resistant cells – at least in part together with the inherent accumulation of these cells in S and G2M phases as revealed from the cell cycle distribution (Figure 5 c) [46].

At this point, it should be emphasized that the ability of US to induce DNA damage - at least under certain conditions - is not a new concept [15], [17], [18]. Being proven, we should expect all post-DNA damage consequences to be possibly occurring after a DNA – damaging US exposure. Recently, it has been reported that DNA-damaging agents could enhance a seemingly omnipresent, newly discovered type of cell division, namely, neosis for self-renewal [31]. Neosis is characterized by the emergence of a progeny of small cells termed “Raju cells”, reaching up to ten, from a single mother cell through budding. In our study, we found that there was an abrupt and significant polyploidy in resistant cells 1 hr post sonication and the microscopic examination 24 hr later also showed nuclear budding and emergence of small cells that was slow and sequential. Generally, the morphological features observed in both cell lines were very similar to that reported for neotic mother cells and the descendant Raju cells reflecting the potential of these cells to undergo leaky neosis especially after acoustic irradiation. Taking into account that “neosis was independent of p53, while the presence of p53 resulted in the death of Raju cells (consistent with the reports that p53 induces apoptosis of cells with genomic instability [31]), one can likewise expect that the degree of tolerance to genetic instability and infidelity in sensitive and drug-resistant cells will determine the fate of the progeny. Therefore, the fluctuating growth ratios of MES-SA cells might have resulted from the eventual death of mother and daughter cells, whereas in case of MES-SA/DX5 cells, increased tolerance to genetic instability, evident from Figure 5 c, could lead to the survival of Raju cells perceived as a steady increase in growth ratios. Interestingly, the growth ratio peaked at D4, a time point similar to that required by HeLa cells (human cervical carcinoma) exposed to ionizing radiation to give off neotic progeny [31]. Neosis as such unveils an alarming aspect of US if applied under conditions inducing DNA damage which, apart from salient (exaggerated) in vitro data, are not far from clinic at least in High Intensity Focused Ultrasound (HIFU) applications [47]. In such case, the risk will not only be restricted to tumor cells but also will include the surrounding milieu for the involvement of neosis in neoplastic transformation. Moreover, neosis may compromise the concept of adjuvant acoustic chemotherapy where enhanced DNA damage is reported [48]. Great care is required in the choice of the order and time of application of dual treatments not only to obtain successful eradication of tumor cells [49], [50], [51], but also to evade the aggravation of tumor growth. In contrast to the significant decrease in viability observed in sequential (Dox-US) protocol, there was “desensitization” of the parent cells to Dox cytotoxicity observed under (US-Dox) sequential-treatment protocol. This desensitization could be justified by Dox - selection of the neotic progeny to give resistant cells. In a similar study, HCT116 human colon carcinoma bearing wild type p53 was forced to senesce by nanomolar concentrations of Dox. When the senescent polyploid cells were allowed to grow in drug - containing medium, the growing Raju cells displayed resistance to a number of antitumor agents [52]. It is worth noting here that the difference in time spans for the emergence of Raju cells between the two studies might be attributable to the different nature of stressors applied as well as to the weakness of the MDR phenotype of MES-SA/DX5 cells which we consider as a favor in our study because it enabled us to detect these changes within the same time frames common in such experiments [53].

Returning to the flow cytometric data, we need to denote that the detection of apoptosis using Annexin V might not be an appropriate method when cells expressing P-gp are used. Pg-p receptors have been shown to act as floppases, translocating a wide range of phospholipids from the membrane inner leaflet [54], [55], in support with the futile hydrolysis (basal ATPase activity) observed for P-gp in absence of exogenous substrates [3]. Therefore, the lack of correlation between cell killing and the percentage of cells binding to Annexin V (Table 4) could be due P-gp activity. To further prove this assumption, cells treated with increasing doses of Dox from 0.5 to 10 µM were analysed after 24 hr-incubation with the drug for DNA fragmentation (according to the method of Sellins and Cohen [18]), and for the extent of binding to FITC-labeled Annexin V flow cytometrically. The results of DNA fragmentation assay showed a clear difference in the sensitivities of both cell lines to Dox (maximum DNA fragmentation % was attained at 1 µM Dox and equals to 23.9±1.7 and 16.1±1.1, for MES-SA and MES-SA/DX5, respectively). Despite this, MES-SA/DX5 cells displayed more PhS redistribution to the external leaflet (data not shown). Generally, the translocation of PhS was 1.5 times higher in the resistant strain whereas it coincided with the parent cell at doses corresponding to changes in Dox-induced cell death modes, namely; senescence, apoptosis and necrosis [56]. Moreover, the assessment of DNA content with PI flow cytometry could be also inappropriate if neosis is taken into consideration. In the study by Bernard et al. [51], the authors observed that the subG1 fraction in US-treated human ovarian carcinoma cells 72 hr post irradiation reached 29% although the microscopic examination of cells indicated that “ultrasound alone had a little effect on inducing apoptosis”. The facts that both the fragmented and condensed DNA exhibit reduced stainability when probed with DNA staining agents [57] and that the genomic content of Raju cells is initially condensed, may justify this discrepancy.

In conclusion, we have shown that US induced intensity-dependent growth inhibition in which DOX-resistant uterine sarcoma cells manifested higher sensitivity to acoustic exposure in agreement with literature. However, DNA-damaging exposures enhanced neotic division shortly after sonication leading to opposite outcomes when Dox was introduced after sonication. The development and selection of resistant progeny by Dox when added after a period sufficient for the initialization of Raju cells emergence reveals serious problems of dual treatment protocols employing DNA damaging agents in general, in which the application time of each treatment relative to the other can be regarded as a key factor in tumor regression or aggression. Future studies should ensue to ensure further understanding of the molecular determinants for induced neosis and to reassess the safety of the multiple acoustic applications based on long term monitoring of cell viability and proliferation.
